# Application of MIR Spectroscopy to the Evaluation of Chemical Composition and Quality Parameters of Foal Meat: A Preliminary Study

**DOI:** 10.3390/foods9050583

**Published:** 2020-05-05

**Authors:** Marta Ruiz, María José Beriain, Miguel Beruete, Kizkitza Insausti, José Manuel Lorenzo, María Victoria Sarriés

**Affiliations:** 1 Research Institute for Innovation and Sustainable Development in Food Chain (IS-FOOD), Universidad Pública de Navarra, Campus de Arrosadía, 31006 Pamplona, Spain; marta.ruiz@unavarra.es (M.R.); mjberiain@unavarra.es (M.J.B.); 2 Multispectral Biosensing Group, Navarrabiomed, Complejo Hospitalario de Navarra (CHN), Instituto de Investigación Sanitaria de Navarra (IdiSNA), Universidad Pública de Navarra (UPNA), Irunlarrea 3, 31008 Pamplona, Navarra, Spain; miguel.beruete@unavarra.es; 3 Centro Tecnológico de la Carne de Galicia (CTC), Rúa Galicia 4, Parque Tecnológico de Galicia, San Cibrao das Viñas, 32900 Ourense, Spain; jmlorenzo@ceteca.net

**Keywords:** MIR spectroscopy, foal meat, chemical composition, quality parameters

## Abstract

The aim of this work was to study the potential of mid-infrared spectroscopy to evaluate the chemical composition and quality parameters of foal meat according to differences based on slaughter ages and finishing diets. In addition, the wavelength ranges which contribute to this meat quality differentiation were also determined. Important characteristics as moisture and total lipid content were well predicted using Mid-Infrared Spectroscopy (MIR)with Rv^2^ values of 82% and 66%, respectively. Regarding fatty acids, the best models were obtained for arachidonic, vaccenic, docosapentaenoic acid (DPA), and docosahexaenoic acid (DHA) with Rv^2^ values over 65%. Quality parameters, as instrumental colour and texture and sensory attributes did not reach high prediction coefficients (*R*^2^). With the spectra data of the region 2198–1118 cm^−1^, samples were accurately classified according to slaughter age (78%) and finishing diet (72%). This preliminary research shows the potential of MIR spectroscopy as an alternative tool to traditional meat chemical composition methods. Finally, the wavelength range of the spectrum from 2198 to 1118 cm^−1^ showed good results for classification purposes.

## 1. Introduction

Nowadays, high assurance of quality and safety during food production is being extremely demanded. Thus, strict controls both throughout the production and during the marketing process are required [1]. This demand and the agricultural industries supervision make highly desirable, food analysis methods which are speed, ease to use, with non-preparation or minimum sample preparation, with non-destruction of samples, low cost, and environmentally sustainable [2,3]. In this way, the Near-Infrared Spectroscopy (NIR) and Attenuated Total Reflectance-Fourier Transform Mid-Infrared Spectroscopy (ATR-FT/MIR) are practical options, as they fulfil the requirements aforementioned. MIR spectroscopy applications in food analysis are diverse although its current use is limited. Especially for meat, several topics have been studied: (1) determination of chemical composition, (2) detection of microbiological spoilage, (3) authentication of products or (4) detection of meat adulterations [1]. Nevertheless, there is a lack of knowledge regarding the use of MIR spectroscopy to evaluate meat quality, concerning not only chemical composition, but also texture, colour parameters, or even sensory attributes. Regarding this last group, Yancey et al. [4] were able to predict the tenderness and overall appraisal evaluated by a consumer panel by the employment of non-invasive techniques such as NIR spectroscopy. In addition, Ripoll et al. [5], obtained successful results predicting some chemical parameters (moisture, fat, water holding capacity), the shear force and sensory tenderness by NIR spectroscopy.

According to world consumption of foal meat, Mongolia (5.81 kg) is the country with the highest horsemeat supply per capita followed by Kazakhstan (4.92 kg), Kyrgyzstan (3.50 kg), Iceland (2.19 kg), Switzerland (0.73 kg) and Italy (0.70 kg) [6]. Currently, studies on foal meat have noticeably arisen because of its healthy properties and sustainable production, which make it an alternative meat for consumers, whose demand for healthy and safe products is continuously increasing [7]. Nevertheless, this kind of meat must be more exhaustively defined according to different factors as slaughter age or feeding, which play an important role in meat quality [8,9]. In this way, rapid, non-destructive techniques such as MIR spectroscopy would be helpful to differentiate foal meat regarding the mentioned factors. Besides, NIR spectroscopy has been widely applied in the food industry, but new wavelengths of the spectrum should be studied in order to try and overcome the current limitations that the near-infrared spectrum has.

Thus, the general objective of the present work was to investigate the potential of MIR spectroscopy to evaluate the chemical composition and quality parameters of foal meat from animals of different ages and feeding regimes. In addition, the wavelength ranges which contribute to this meat quality differentiation were also determined.

## 2. Materials and Methods

### 2.1. Animal Management and Meat Sampling

Forty-six foals obtained by crossing Galician Mountain mares with a Burguete stallion were used. The animals were reared under semi-extensive conditions. A fuller description of the animal management has been published in Ruiz et al. [10]. Twenty-two foals were slaughtered at 13 months (13M) and another 24 at 26 months of age (26M). Prior to slaughter, all the foals were supplemented for a fattening period of about 104 days. Two study groups were formed by randomly assigning 11 foals from the 13M group and 12 foals from the 26M group to be supplemented on pasture with a standard concentrate (SC) (2 kg per foal and day) and pasture; and 11 foals from the 13M group and 12 foals from the 26M group to be supplemented on pasture with a linseed-enriched concentrate (5%) (LC) (2 kg per foal and day) and pasture.

Foals were slaughtered and dressed according to the specifications outlined in the European legislation (Council Regulation 1099/2009).

Immediately after slaughter, hot carcass weight was determined, and once chilled for 24 h at 4 ± 1 °C, cold carcass weight (kg) and dressing percentage (%) were determined 10]. At this point, the left half-carcasses were transported under refrigeration to the research center pilot plant at 4 ± 1 °C. *Longissimus thoracis* (LDT) muscle was removed from the left half-carcasses. This muscle was employed because it is the reference muscle in meat science studies, and it is one of the most commercially valuable muscles with respect to foal meat. Twenty-four hours after removal, 46 LDT samples were analysed. In order to carry out the analyses, the LDT section ranging between the seventh and the twelfth rib was employed, following the same cutting order for all the analyses. They were then sliced into five 20 mm (±0.2) thick steaks (FIRMAQ, V-900, Lorca, Spain) simulating the standard cutting thickness at the sale point. Then, they were vacuum-packaged and frozen at −18 °C (±2) to be transported to the Public University of Navarra where samples were frozen-preserved for ninety days and then, prior to each analysis, samples were thawed overnight in a fridge at 4 + 1 °C.

### 2.2. Meat Physicochemical Analysis

#### 2.2.1. Chemical Composition

The moisture, protein, ash, total lipids content, pH, and water holding capacity were performed following the procedure of Dominguez et al. [11]. Total and soluble collagen content were determined from the hydroxyproline content [12].

#### 2.2.2. Meat Colour Evaluation

The spectral reflectance of the samples, provided by a Minolta CM2002 spectrophotometer with a D65 illuminant and a 10° standard observer, was used to calculate the proportion of each pigment form. Relative myoglobin (DMb) (%), metmyoglobin (MMb) (%) and oxymyoglobin (OMb) (%) contents were obtained from the reflex attenuance at the isobestic points 572, 525, 473, and 700 nm [13]. Reflectance values were converted into absorbance values according to the formula:

A = 2−log10R(1)

where A is absorbance and R is reflectance [14].

To assess meat colour by Image Analyses, RGB colour values were determined for each sample (R, G, and B) individually. Where R stands for red colour, B means blue, and G stands for green. The colour range varies from 0 to 225. The value “0” means: 100% of the light is reflected, and value “225” means: 100% of the light is absorbed. Samples were examined under the microscope and both sides photographed. The microscope images obtained were digitised by the employment of an image analysis software (ImageJ 1.47, LOCI, University of Wisconsin, WI, USA). The average red, green and blue values were obtained. The CIEL*a*b* system was also employed. Lightness (L*), redness (a*), yellowness (b*), chromaticity (C*) and hue (h*) [15] were assessed following the methodology established by Mendizabal et al. [16].

#### 2.2.3. Intramuscular Fatty Acids Analysis

Fatty acid profile was determined as it is shown in Domínguez et al. [14]. But, for this study, the most remarkable fatty acids from the nutritional point of view were selected. These are: stearic (C18:0), oleic (C18:1n9c), linoleic (C18:2n6c), linolenic (C18:3n3), arachidonic (C20:4n6) and vaccenic (CLA precursor) (C18:1n11t) acids. Finally, eicosapentaenoic (EPA, C20:5n3), docosapentaenoic (DPA, C22:5n3), docosahexaenoic (DHA, C22:6n3) and n-6 total PUFA content were studied as well.

#### 2.2.4. Texture Analysis

The maximum shear force was assessed using a Warner–Bratzler shear force (WBSF) (N) device. Eight replicates per sample of 1 cm^2^ (square cross-section), with muscle fibres parallel to the longitudinal axis of the sample were analysed. A TA-XT2i texturometer (Stable Micro Systems, Surrey, United Kingdom) was used connected to an IBM-compatible Foxen computer, with microprocessor AutenticAMD-K6™ 3D processor. The program employed was “Texture Expert” version 1.22 to Windows (Stable Micro Systems, Surrey, UK).

### 2.3. Consumer Panel Evaluation

A consumer panel integrated by 247 panelists from Pamplona (Navarra, Spain) was employed. The demographic distribution of consumers for the experiment was as follows: according to gender (50.3 vs. 49.7% for male and female, respectively), age (36.9 vs. 35.2 vs. 19.7 vs. 8.3%, for age ranges of 20–34, 35–50, 51–65, and >65 respectively), and education (73.2 vs. 20.5 vs. 6.3%, for degree, secondary, and elementary studies, respectively) and incomes (3.2 vs. 12.1 vs. 45.0 vs. 36.0%, for income ranges of < 900€/month, 900€–1.500€/month, 1.500€–3.000€/month, and >3.000 €/month, respectively). The methodology used for consumer taste panels was previously described by Beriain et al. [17]. Nine sessions integrated by 27/28 consumers per session took place. The frozen steaks were thawed at 2 °C for approximately 24 h and cooked on a grill (Magefesa, Spain) according to the AMSA guidelines [18] until they reached 70 °C of internal temperature. Four steak-pieces (one from each age and diet combination) were presented to the consumers one by one in a random order [19]. Panelists were asked to rate their liking for tenderness, juiciness, and their overall appraisal of the samples. A 9-point hedonic scale was used, being 1 = “Dislike extremely”, 2 = “Dislike very much”, 3 = “Dislike moderately”, 4 = “Dislike slightly”, 5 = “Neither like nor dislike”, 6 = “Like slightly”, 7 = “Like moderately”, 8 = “Like very much” and 9 = “Like extremely”.

Statistical analysis was conducted using the IBM SPSS Statistics 25 for Windows (SPSS Inc. Corporation, NY, USA) to obtain the descriptive statistics of the physical, chemical, and consumer evaluation of the samples. The mean plus standard deviation and the coefficient of variation (%) were obtained.

### 2.4. Mid-Infrared Spectra Measurements and Spectral Acquisition

All the samples were thawed overnight in a fridge at 4 ± 1 °C, prior to the analysis with the ATR-FT/MIR spectroscopy. The measuring instrument used in this research was a FTIR Vertex 80v spectrometer (Bruker, Ettlingen, Germany). This equipment allows to work under vacuum conditions in the optical system which reduces the possible interferences produced by the water vapor or the carbon dioxide in the measurements. This device is located in a clean room certified according to ISO 14644-1:2015. There, the concentration of particles present in the air and the temperature (constantly maintained at 22 °C) were controlled. The measurements were made with an accessory A225 / QPlatinum-ATR (Bruker, Germany) made of a diamond crystal.

Firstly, a reference spectrum was taken with the ATR device empty, and then the spectrum of each of the samples was measured. It was necessary to place the meat sample on the crystal, ensuring the entire crystal surface was completely covered and that there was a perfect contact between the sample and the crystal. In total, 6 replicates were performed per sample. For each sample, 32 scans in the 4000–400 cm^−1^ spectral range were recorded with a resolution of 4 cm^−1^.

### 2.5. Selection of Optimal Wavenumber Region and Spectral Pre-Treatment Method

Different wavenumber ranges and pre-treatment methods (first/ second derivative, standard normal variate, maximum/ minimum or vector normalisation, multiplicative scatter correction or removal of constant slope) were studied to find models with better performance. These pre-treatments were employed on the performance of PLS as a previous step before being evaluated. The selection of pre-treatments and spectral ranges supplied by models represents a key parameter to be taken into account in a multivariate analysis according to a specific program of chemometrics, OPUS Quant 2 (Bruker, Ettlingen, Germany).

### 2.6. Data processing and Calibration Models

#### 2.6.1. Regression Model

Partial least squares (PLS) regression is a supervised analysis based on the relation between spectral intensity and sample characteristics [20]. In the present study, PLS technique was used to obtain quantitative prediction of the parameters of interest and it is generally carried out in two steps. To construct a model, the first step is to collect a calibration set of samples for establishing a multiple linear regression between the Mid-Infrared Region (MIR) spectra and the various parameters of the sample set in order to perform a calibration. The second step consists in testing developed calibration models on validation sets so as to verify model accuracy and robustness.

The chemical bonds associated with each peak of the FTIR spectra were determined by analyzing the physicochemical composition and sensory properties of the samples, by correlations among the bands with larger intensity, and by comparing the wavenumbers with the literature [3].

Then, the analysis was particularised to the physical, chemical, and sensory properties of the samples, and prediction models were built using the results obtained by the described methods as reference values. Spectral data pre-treatments such as standard normal variate (SNV), multiplicative scatter correction (MSC) and first or second order derivatives were applied to the spectra to reduce scattering effects and to correct peak overlap and baseline drifts. The models were built using a specific program of chemometrics, OPUS Quant 2 (Bruker, Ettlingen, Germany).

#### 2.6.2. Validation Model

The calibration models were developed using the Partial Least Square (PLS) regression method and validated by cross-validation. The main advantage of cross-validation is that a small number of samples are required because the same set of samples is used to calibrate and validate the method. The optimum number of factors in the PLS calibration models was indicated by the lowest number that gave the minimum value of the root mean square error (RMSE) in cross-validation, in order to avoid overfitting of the models. In each variable analysed, the range of wavenumbers with more information in their absorbance and the best pre-treatment method were selected using the OPUS Quant 2 program. During the calibration and prediction stages, the outliers were detected and kept out of the prediction models. The models were tested to predict the different parameters in the independent validation set and the best calibration models were selected based on the highest determination coefficient of calibration (Rc^2^), determination coefficient of cross-validation (Rv^2^) and the lowest root mean square error of calibration (RMSEC), root mean square error of cross-validation (RMSECV) and the ratio of prediction to deviation (RPD). These models enable a quantitative estimation of the physicochemical composition, colour parameters, and sensory attributes of the different samples depending on the absorbance intensity.

### 2.7. Multivariate Analysis

#### 2.7.1. Pearson’s (*r*) coefficients of correlation

Pearson’s (*r*) coefficients of correlation between the meat quality response variables were determined. In addition, correlations between the most relevant wavelengths found throughout the spectra were studied. A positive correlation coefficient close to 1 indicates that when band intensity increases, the signal of the other band increases as well. This means that the same link or structural unit is in different bands with different modes of vibration. A negative correlation coefficient nearby to −1 reflects that an increase in a signal coincides with the decrease in the other compared signal. In no case, correlation must be understood as synonymous with causality.

#### 2.7.2. Principal Component Analysis (PCA)

With the most representative wavelength range obtained by the models, principal component analysis (PCA) was applied to reduce the number of variables. PCA determines linear combinations of the original variables to summarise the data with minimal loss of information. Varimax rotation was applied to the factors to facilitate interpretation and maximise the explained variance. Once the PC variables were obtained, canonical discriminant analysis was carried out to classify the samples. A stepwise model analysis using a significance level of 0.05 as the variable entry criterion was applied for the discriminant procedure. The leave-one-out cross-validation method was used to validate the model. 

#### 2.7.3. Canonical Discriminant Analysis

Canonical discriminant analysis method was developed to classify the animals into the different slaughter ages and finishing diets. Discriminant analysis was developed including the medium spectroscopy wavelengths, and it was conducted using stepwise model analysis, which performed the best-subset selection of the quantitative predictor by a procedure of entrance-remove of variables in the model. The significant level of a variable to enter in the model was 0.05.

## 3. Results

### 3.1. Physicochemical and Sensory Description of Longissimus Thoracis Et Lumborum Muscle of Foal Meat

Table 1 shows a description of foal meat in terms of composition and quality according to the slaughter age (SA) and finishing diet (FD). No interactions (SAxFD) were found (*p* < 0.05) on any of the parameters evaluated, except for ash, L*, b*, C*, h* and WHC as reported by Domínguez et al. [11] in these same animals. In terms of chemical composition, the most noteworthy point is the lower moisture and more than twice the total lipids content presented by the 26M samples in contrast to the 13M samples (*p* < 0.001; *p* < 0.001; respectively). Fatty acids are generally different depending on slaughter age (*p* < 0.001), but not on finishing diet (*p* > 0.05). According to RGB values, all of them were higher in 26M and LC than in 13M samples and SC samples (*p* < 0.001; respectively). Regarding CIELab colour space, the 26M samples showed lower L* (*p* < 0.001) and higher a* (*p* < 0.001) and C* values (*p* < 0.001) than the 13M samples. With regard to the FD effect, the SC samples seemed to have higher L* and lower a*, b* values than the LC samples (*p* < 0.01; *p* < 0.01; *p* < 0.05, respectively). Regarding WBSF, the 26M samples presented higher shear force (N) than the 13M samples (*p* < 0.05). As for the FD effect, the meat from the SC group showed higher values of shear force (N) than that from the LC group (*p* < 0.05). Sensory attributes evaluated by consumers showed that meat from 13M foals showed higher tenderness (*p* < 0.001) and juiciness (*p* < 0.05) values than 26M samples. Thus, it can be stated that the differences due to finishing diet were less relevant than the differences due to slaughter age. 

Regarding the relationship between physicochemical and sensory variables, the most remarkable correlations were found between the overall appraisal with tenderness and juiciness (*r* = 0.89, *r* = 0.87, respectively; *p* > 0.01). Moisture was positively correlated to tenderness (*r* = 0.38; *p* > 0.01) and overall appraisal (*r* = 0.31; *p* > 0.05), whereas total lipids content was negatively correlated to tenderness (*r* = −0.40; *p* > 0.01) and juiciness (*r* = −0.35; *p* > 0.05). All colour variables from the image analyses (RGB) and the CIELab system were negatively correlated to overall appraisal (*p* > 0.05).

### 3.2. Calibration and Validation Models to Predict the Chemical Composition and Quality Parameters of Foal Meat

In order to look into the intrinsic properties of foal meat, regression models were built based on the two spectrum ranges (3200–2500 cm^−1^ and 2300–980 cm^−1^) to estimate the chemical composition and quality parameters of the samples analysed from the spectral information.

**Figure 1:**
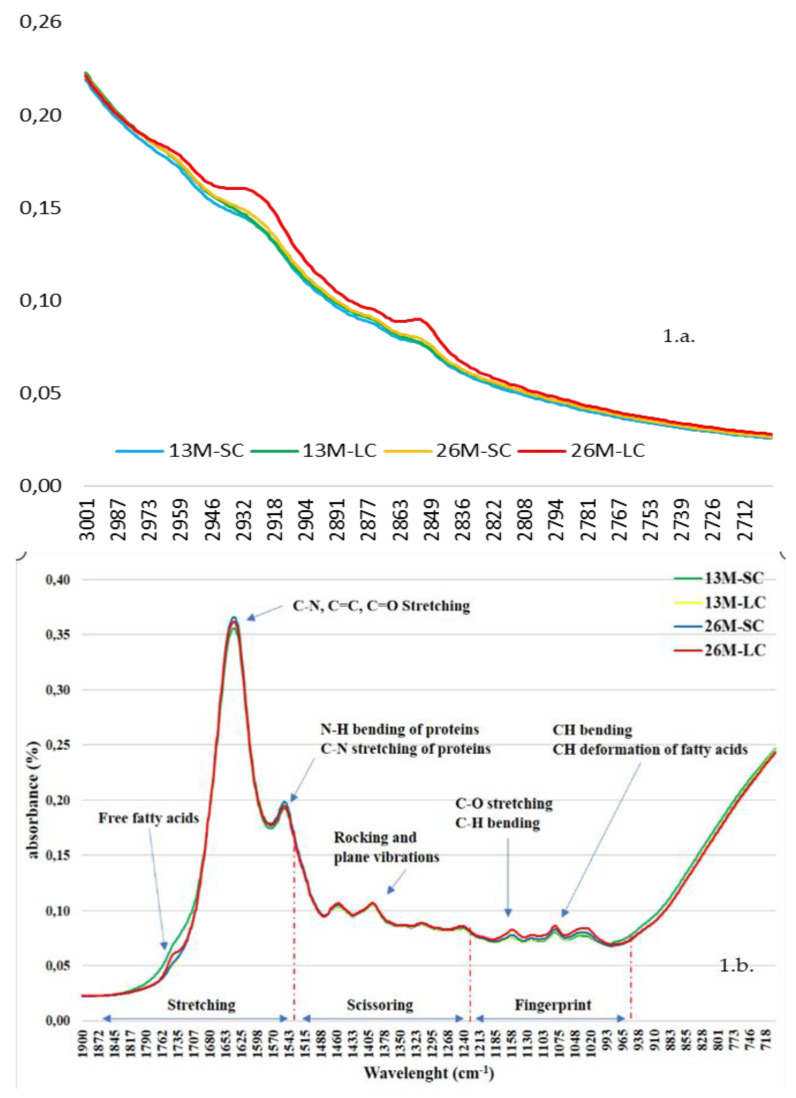
Spectral characteristics of the samples from 13- and 26-month-old foals (13M, 26M) and supplemented with standard and linseed concentrate (SC, SL). Range selected from 3000–2700 cm^−1^ (**a**) and from 1900–750 cm^−1^ (**b**) according to Lozano et al. [3].

Figure 1 shows the typical spectrum characteristics obtained by FT-MIR analysis in samples from 13 and 26-month-old foals (13M, 26M) and supplemented with standard and linseed concentrate (SC, SL). Figure 1a, represents the section from 3000 cm^−1^ to 2700 cm^−1^ and Figure 1b represents the region from 1900 cm^−1^ to 750 cm^−1^.

Based on the obtained spectrum data, Table 2 shows mathematical treatments and wavelengths for each calibration and validation equation according to chemical composition, fatty acid profile, and quality parameters, respectively.

In Table 2, it is shown that moisture and total collagen showed coefficient of determination for validation (Rv^2^) higher than 70%, Total lipids content reached higher coefficient of determination for validation (Rv^2^) (66%) (Rc^2^) compared to calibration (76.30%). With regard to pigment content (%), oxymyoglobin (OMb), deoxymyoglobin (DMb), and metmyoglobin (MMb) reached low validation coefficients (Rv^2^<26%). Nevertheless, the three of them seemed to be related to, at least, the wavelength range 1839–1478 cm^−1^. Finally, the rest of the variables did not reach 26%, except for ash content (40.55%). According to the fatty acids, Rv^2^ for linoleic, linolenic, EPA and omega-3 polyunsaturated fatty acids did not reach 60%, in spite of reaching Rc^2^ values over 88%. The best prediction models, with Rv^2^ over 75%, were obtained for arachidonic, DPA, DHA (with RPD values over 2), and omega-6 polyunsaturated fatty acids (with an RPD value close to 2). The most repeated selected wavelength ranges were 3838–3277, 2919–2558, and 1839–1118 cm^−1^. The best treatments were first derivate plus standard normal variate (SNV) and multiplicative scatter correction (MSC).

Regarding colour, RGB variables reached high values for Rc^2^ (over 90%) and Rv^2^ (over 60%, with the exception of red), whereas the most commonly studied colour coordinates (L*, a*, b*, C*, h*) reached both calibrations and validation coefficients below 40% and 22%, respectively, being h* the worst predicted variable (4.49%). The selected wavelength ranges for RGB colour variables were 3998–3637 and 2198–1118 cm^−1^, and the first derivate + MSC was used as prediction treatment for most of the cases. On the other hand, no enclosed wavelength range were described for L*, a*, b*, C* and h*.

For WBSF, the prediction results were poor (Rv^2^=25.70%). Likewise, predictability for all sensory attributes were poor with the highest Rv^2^ and Rc^2^ values being 27.21% and 36.17%, respectively. The three sensory variables were negatively correlated to WBSF (tenderness; *r*=−0.82, *p* < 0.01; juiciness; *r*=−0.89, *p* < 0.01; overall appraisal; *r*=−0.66, *p* < 0.05) and the low accuracy of WBSF prediction could be reflected in the sensory attributes. Besides, WBSF was positively correlated with cooking losses [11], and meat moisture was also highly and negatively related to intramuscular fat content (*r*=−0.89; *p* < 0.01).

### 3.3. Principal Component and Discriminant Analyses

After the results obtained from the chemometric analysis, three different wavelengths ranges were selected according to the prediction accuracy for chemical composition, the studied fatty acids, and the quality parameters. These were: 3278–2918 cm^−1^ for the chemical composition variables group, 2919–2558 cm^−1^ for the fatty acids group and 2198–1118 cm^−1^ for the quality parameters group. Taking these ranges into account, canonical discriminant analyses were again carried out in order to improve the first classification obtained before developing the calibration and validation models (Table 3). From these results, the best wavelength range in order to classify samples according to slaughter age and finishing diet is the one which better predicts the quality parameters: 2198–1118 cm^−1^. These results showed that for slaughter age, samples were classified with an accuracy around 78.3% whereas regarding finishing diet, samples were classified with an accuracy around 71.8%. Even if just one classification result was higher than 80%, there are three results close to 80% (77.3%, 79.2%, and 78.3%) that make MIR an interesting alternative method.

## 4. Discussion

Regarding the spectra characteristics of the samples in the medium infrared, Lozano et al. [3] described the two most important spectra ranges as far as foal meat is concerned: 3200–2500 cm^−1^ and 2300–980 cm^−1^. These two sections are shown in Figure 1a and in Figure 1b for meat samples from 13M and 26M foals supplemented with SC and LC concentrate samples (13M-SC, 13M-LC, 26M-SC, 26M-LC). Both ranges have been slightly widened not to lose possible wavelength which could show some differences between each group. The region from 3000 cm^−1^ to 2700 cm^−1^ (Figure 1a) is the one of hydrogen’s stretching related to -C-H stretching vibration, involving double bounds =C-H and aliphatic CH_3_ and CH_2_ [21,22]. It is shown that 26M-LC samples described higher absorbance peaks than 13M-SC/LC and 26M-SC between 2990 and 2800 cm^−1^. This means that asymmetric and symmetric stretching vibrations of -C-H of aliphatic CH_3_ at 2959 and 2873 cm^−1^ were taking place more intensely than in the other groups. Moreover, the absorbance difference at 2925 and 2851 cm^−1^ (peaks related to asymmetric and symmetric stretching vibrations of -C-H of aliphatic CH_2_) between 26M-LC and the others 3 groups, was even higher, and thus more intense. Domínguez et al. [11] showed that 26M foals’ samples and those from foals supplemented with linseed concentrate presented a higher amount of monounsaturated fatty acids and reached higher total lipids content (Table 2). This higher amount of aliphatic CH_3_ and CH_2_ compounds define larger fatty acids chains, indirectly represented with a higher absorbance.

The region showed in Figure 1b, involves double bond’s stretching (1750–1650 cm^−1^), other deformations and bendings (1500–1200 cm^−1^) and the called “fingerprint range” (1200–950 cm^−1^) [3,21,22]. The 13M-SC samples, which presented a higher percentage of saturated and polyunsaturated fatty acids (35.2 g/100 g vs. 33.4 g/100 g) [14] and higher lipid oxidation than the rest of groups (0.40 vs. 0.36 mg MDA/kg fresh meat) (data not shown), showed a higher absorbance from 1800 to 1700 cm^−1^. Bands at 1742 cm^−1^ are related to triglycerides and free fatty acids. Vlachos et al. [23] explained that an oxidation of fatty acids is likely to happen close to 1700 cm^−1^, and that the accurate peak position and intensity would depend on the fatty acid composition. Thus, it might be possible that the higher absorbance obtained for 13M-SC samples, was linked to the high amount of polyunsaturated fatty acids and a possible lipid oxidation.

Bonds of N-H, C=C, C-N, and the combination of N-H with C-H (Amides II) are typical of protein amino acids and appear at bands 1657 and 1542 cm^−1^ [3]. Figure 1 does not show any difference in the absorbance although it seems to be slightly higher in 26M-SC foal meat samples. Along the fingerprint range, a smooth absorbance increase can be seen in the 26M-LC foal meat samples, but not determinant. This region is related to stretching vibrations of the C-O bond stretching vibration and the C-H bond bending vibration and the peak at 1117 cm^−1^ is assigned to bending and twisting vibration of the fatty acids [24]. Correlations help to better understand the bands’ changes due to different vibrations. The absorbance ~ 2959 cm^−1^ was positively correlated to the peak ~2925 cm^−1^ (0.78), ~2837 cm^−1^ (0.94), ~2851 cm^−1^ (0.74), ~1658 cm^−1^ (0.81), ~1542 cm^−1^ (0.67) and ~1117 cm^−1^ (0.73). And these wavenumbers are related to typical vibration modes of the lipids’ fatty acids and proteins (C-H bonds). A weak and negative correlation between 2959 and 1742 cm^−1^ was found (−0.38**). This means that the stretching vibration of carbonyl bond of esters and free fatty acids [3] related to this peak is different from the vibration way at 2959 cm^-1^. Another example of different vibration is better understood by the positive correlation found between the peak at ~1117 cm^−1^ and ~2925 (0.88**), ~2873 (0.88**) and ~2851 cm^−1^ (0.90**). All these peaks are related to vibration of the fatty acids. But ~1117 cm^−1^ is assigned to bending and twisting vibration whereas the other ones are related to stretching vibrations.

The lack of MIR spectroscopy studies dealing with meat composition and meat quality makes necessary the comparison of the obtained results with studies of NIR spectroscopy, although the spectra range is not the same. It should be explained that the ratio of prediction to deviation (RPD) is noticeably poor with regard to validation results, except for moisture (2.33). High quality moisture models have been reported in the literature, with coefficients of determination higher than 0.70 in NIR [5] This means that the regression models have a low accuracy to predict different chemical composition parameters of foal meat. The prediction model for protein had a low Rv^2^ (22.71%). Difficulties in predicting meat protein content have been previously reported in beef [6,25] with NIR spectroscopy. One of the causes of the low accuracy of protein prediction in meat could be related to the analytical differences between the Kjeldahl determination (which measures nitrogen) and the MIR/NIR techniques (which measures protein bonds) [26]. Another reason could be related to the MIR spectra record fundamental molecular vibrations, which can be more easily affected by multiple interferences [27]. Nevertheless, better results were obtained when applying MIR to the prediction of fat and protein in beef [3]. On the contrary, when comparing the results with Ripoll et al. [5] similar results were observed for chemical composition with NIR spectroscopy. This fact could be due to the more developed beef production systems compared to foal production systems, which could favour better regression results. For both moisture and protein content, the maximum and minimum normalisation treatment were used, being 1839–1478 cm^−1^ and 1119–759 cm^−1^ the wavelength ranges employed.

Higher validation values were reported in beef (76%) [5] or lamb (73%) [28] with NIR spectroscopy. This could be due to the fact that measurements were taken on the intact sample instead of on a homogenised sample. Total collagen content showed also high Rv^2^ (70.65%), far from those reported by other authors with NIR spectroscopy in beef [29]. For both total lipids and total collagen content, it must be mentioned that RPD values (1.72 and 1.85, respectively) were close to 2, threshold which defines an acceptable regression model. In addition, in both cases the wavelength selected was 1479–1118 cm^−1^. These models are in concordance with the obtained spectra results and with the literature mentioned according to fatty acids [23,24]. Therefore, these results are of great practical importance and they make in evidence the usefulness of MIR spectroscopy technique and the need of gaining deeper knowledge on this research field. It must be mentioned as well, that the vaccenic acid (precursor of conjugated linoleic acid, CLA), reached Rv^2^ of 67.11% and an RPD value of 1.74. The potential properties of CLA for human health [30] support the need of developing further studies to be able to detect it, qualitative and quantitatively.

The lack of references dealing with the estimation of colour parameters with both NIR and MIR spectroscopy makes a deeper discussion of the results more difficult.

Regarding meat texture, as stated by Ripoll et al. [5], the strong relationships between moisture, fat, and cooking losses with WBSF could explain the WBSF behaviour better than its own calibration model. Geesink et al. [31] did not obtain useful models for WBSF, owing to limited muscle variance (CV: 21%). This fact could be another reason for the weak prediction result in the present research (ranged 10–14%).

As reported by Ripoll et al. [5] and Andrés et al. [32] for sensory traits in beef and lamb respectively, tenderness, juiciness, and overall appraisal are hard to estimate as they are subjective judgements and their correlations to other instrumental determinations are weak. The low prediction values obtained for most of the quality parameters and consumer evaluation results could be explained by the low number of samples employed. Perhaps, these prediction models would be better with a higher number of samples [3]. Besides, foal meat is characterised by its heterogeneity, and, what is more, it suffers rapid internal composition changes [10] that could highly affect the spectra results. These results could be also due to the interferences related to the homogeneity and particle size of the sample and its performance [33].

After applying discriminant analysis based on the spectra ranges 3200–2500 cm^−1^ and 2300–980 cm^−1^ for classifying the samples according to slaughter age or finishing diet, the obtained results showed lower values in the present study than those found by Xing et al. [34] in intact pork meat (85% accuracy) and Juárez et al. [35] who classified six sheep breeds (83% accuracy) by visible spectroscopy. These results may let infer that the variables obtained from the multivariate statistical treatment, which involved the most relevant spectra data, are mainly related to different characteristics due to the slaughter age than due to finishing diet. Moreover, according to the relationship appreciated in the spectra between different wavelengths and the fatty acids, it could be highlighted that the total lipid contents would be one of the most evident causes why 13M and 26M foals are different.

## 5. Conclusions

The MIR spectra obtained in the present research showed some remarkable differences mainly due to the fat and the fatty acid composition. Prediction models were accurate for moisture, collagen, and lipids, most of the fatty acids (chemical composition) and for the RGB colour coordinates (quality parameters). Classifications by discriminant analysis showed good results after the use of regression treatments delimiting the range of the spectrum to 2198–1118 cm^−1^ and coinciding with the wavelengths in which a large number of protein and fatty acid bonds are recognised.

From a commercial point of view, and based on the obtained results, the use of MIR spectroscopy could be used to sort out meat from “old vs young” foals. Less precision was achieved according to the foals feeding. Anyway, due to the low accuracy to predict different chemical composition and sensory parameters of foal meat, and considering the potential of this technique, more studies are needed in order to improve the estimation of meat characteristics.

Since the present study intends to be a preliminary approach to know the possibilities of the application of MIR spectroscopy to the study of meat quality, for future researches it would be necessary to use a larger sample number and study other physical characteristics such as protein denaturation or sarcomere length which could help to understand changes in the bonds.

Summarising, MIR spectroscopy could be a potential alternative to traditional meat composition analytical methods but, at the present study, the obtained accuracies are low for industry application and still need to be improved.

## Figures and Tables

**Table 1:** Physicochemical and sensory description of *Longissimus thoracis et lumborum* muscle from Galician Mountain x Burguete crossbred foals slaughtered at 13 and 26 months of age (13M, 26M) and supplemented with standard and linseed concentrate (SC, LC) (*n* = 46). Mean plus standard deviation and coefficient of variation (CV%).

	Slaughter Age				Finishing Diet			
	13M	CV%	26M	CV%	SC	CV%	LC	CV%
Chemical composition								
Moisture (%)	74.5 ± 0.15	0.2	72.6 ± 0.28	0.4	73.9 ± 0.27	0.4	72.1 ± 0.33	0.5
Protein (%)	22.5 ± 0.19	0.8	22.7 ± 0.19	0.8	22.6 ± 0.18	0.8	23.2 ± 0.21	0.9
Ash (%)	1.26 ± 0.02	0.0	1.38 ± 0.03	2.4	1.30 ± 0.03	2.3	1.17 ± 0.03	2.4
Total lipids content (%)	0.37 ± 0.08	14.0	1.82 ± 0.16	9.3	0.74 ± 0.22	18.3	1.61 ± 0.14	12.1
Total collagen (g/100 g meat)	0.36 ± 0.04	11.1	0.44 ± 0.05	11.4	0.40 ± 0.01	2.5	0.40 ± 0.11	27.5
Soluble collagen (%TCa)	4.11 ± 0.58	14.1	2.39 ± 0.63	26.4	3.40 ± 0.87	25.6	3.11 ± 0.87	28.0
pH	5.61 ± 0.02	0.4	5.66 ± 0.02	0.4	5.56 ± 0.02	0.4	5.58 ± 0.02	0.4
Water Holding Capacity	23.3 ± 0.61	2.8	22.7 ± 0.44	1.9	20.2 ± 0.41	1.8	22.7 ± 0.60	2.8
Deoxymyoglobin (DMb) (%)	25.1 ± 5.11	20.4	22.4 ± 13.44	60.0	23.2 ± 8.64	37.2	24.3 ± 7.84	32.3
Metmyoglobin (MMb) (%)	16.9 ± 9.63	57	22.0 ± 5.91	26.9	18.9 ± 3.23	17.1	20.0 ± 5.62	28.1
Oxymyoglobin (OMb) (%)	58.0 ± 8.14	14.0	55.7 ± 11.21	20.1	57.9 ± 7.84	13.5	55.8 ± 9.69	17.4
Fatty acids (g/100 g)								
Stearic acid	7.21 ± 0.20	2.8	5.10 ± 0.19	3.7	6.12 ± 0.30	4.9	6.09 ± 0.29	4.8
Oleic acid	23.0 ± 1.03	4.5	30.8 ± 0.88	2.9	25.5 ± 1.39	5.5	28.6 ± 1.03	3.6
Linoleic acid	18.4 ± 0.85	4.6	11.9 ± 0.74	6.2	15.9 ± 1.16	7.3	14.1 ± 0.88	6.2
Linolenic acid	11.1 ± 0.37	3.3	11.9 ± 0.59	5.0	11.1 ± 0.53	4.8	11.8 ± 0.48	4.1
Arachidonic acid	1.73 ± 0.12	6.9	0.99 ± 0.08	8.1	1.48 ± 0.14	9.5	1.20 ± 0.11	9.2
Vaccenic acid	0.05 ± 0.01	20.0	0.03 ± 0.00	0.00	0.04 ± 0.01	25.0	0.04 ± 0.00	0.0
Eicosapentaenoic acid (EPA)	0.97 ± 0.08	8.3	0.37 ± 0.03	8.1	0.73 ± 0.09	12.3	0.58 ± 0.07	12.1
Docosapentaenoic acid (DPA)	1.81 ± 0.10	5.5	0.92 ± 0.07	7.6	1.48 ± 0.14	9.5	1.21 ± 0.12	9.9
Docosahexaenoic acid (DHA)	0.48 ± 0.03	6.3	0.21 ± 0.02	9.5	0.38 ± 0.04	10.5	0.29 ± 0.03	10.3
Total n-3 Polyunsaturated fatty acids (PUFAs)	14.9 ± 0.35	2.4	13.8 ± 0.60	4.3	14.2 ± 0.58	4.1	14.4 ± 0.45	3.1
Total n-6 Polyunsaturated fatty acids (PUFAs)	21.1 ± 1.00	4.7	13.6 ± 0.84	6.2	18.3 ± 1.35	7.4	16.1 ± 1.03	6.4
Quality parameters								
Red	141.6 ± 6.93	5.0	161.9 ± 9.63	6.0	150.6 ± 17.89	11.9	152.9 ± 7.42	4.9
Green	85.9 ± 6.73	7.8	98.7 ± 5.54	5.6	90.3 ± 9.71	10.8	94.3 ± 4.15	4.4
Blue	99.0 ± 8.88	8.8	112.9 ± 7.39	6.5	103.2 ± 11.81	11.4	108.6 ± 5.43	5.0
L* (Lightness)	32.49 ± 2.31	7.1	28.90 ± 3.25	11.3	31.82 ± 2.99	9.4	29.57 ± 3.36	11.4
a* (Redness)	17.59 ± 1.32	7.5	20.35 ± 3.15	15.5	17.89 ± 1.85	10.3	20.05 ± 3.17	15.8
b* (Blue)	10.01 ± 1.67	16.7	11.49 ± 3.81	33.2	9.92 ± 1.83	18.5	11.58 ± 3.74	32.3
C (Chroma)	20.29 ± 1.55	7.6	23.46 ± 3.51	15.0	20.52 ± 2.03	9.9	23.23 ± 4.53	19.5
h (Hue angle)	29.56 ± 4.25	14.4	28.79 ± 4.98	17.3	28.94 ± 4.72	16.3	29.41 ± 4.58	15.6
Warner–Bratzler shear force (WBSF) (Newtons)	45.88 ± 5.73	12.5	53.27 ± 5.71	10.7	52.71 ± 4.99	9.5	46.44 ± 6.73	14.5
Tenderness	5.76 ± 0.64	11.1	5.03 ± 0.88	17.5	5.44 ± 0.60	11.0	5.35 ± 0.55	10.3
Juiciness	5.61 ± 0.63	11.2	5.14 ± 0.85	16.5	5.45 ± 0.63	11.6	5.31 ± 0.74	13.9
Overall appraisal	5.65 ± 0.45	8.0	5.36 ± 0.61	11.4	5.60 ± 0.53	9.5	5.41 ± 0.69	12.8

Some of the data were previously reported by Domínguez et al. [11] (License Number 4811250281534).

**Table 2:** Results of the PLS regression models for the MIR data matrix of meat composition and quality from 13- and 26-month-old Galician Mountain x Burguete crossbred foals (13M, 26M) supplemented with standard and linseed concentrate.

			Calibration			Validation				
	*n* ^a^	*p* ^b^	Rc^2^ (%) ^c^	RMSEC ^d^	RPD	Rv^2^ (%) ^e^	RMSECV ^f^	RPD ^g^	Treatment	Selected Regions (cm^−1^)
Chemical Composition										
Moisture	41	9	93.67	0.34	3.97	81.57	0.53	2.33	Max. and min. normalisation	3278–2918; 1839–1478; 1119–759
Protein	42	1	30.36	0.75	1.20	22.71	0.78	1.14	Max. and min. normalisation	3998–3637; 1839–1478; 1119–399
Ash	42	5	97.92	0.02	6.36	40.55	0.09	1.30	2nd derivate	3278–2918; 2198–1838
Total lipids content	38	5	26.30	0.85	1.16	65.99	0.46	1.72	2nd derivate	2559–1838; 1479–1118
Total collagen	40	8	98.12	0.01	7.39	70.65	0.04	1.85	1st derivate + MSCh	3638–3277; 1479–1118
Soluble collagen	45	1	9.78	2.67	1.05	0.615	2.72	1.00	MSC	2198–1838
pH	40	1	31.19	0.07	1.21	22.39	0.07	1.14	Removal of constant slope	1479–1118
Water Holding Capacity (WHC)	37	1	29.88	1.56	1.19	20.03	1.60	1.13	Removal of constant slope	759–399
Deoxymyoglobin DMb	46	10	97.88	6.88	1.55	25.78	8.02	1.16	2nd derivate	3278–2918; 1839–1118
Metmyoglobin (MMb)	45	3	35.31	5.21	1.24	21.63	5.48	1.13	1st derivate + MSC	1839–1478
Oxymyoglobin (OMb)	46	3	29.23	8.27	1.19	16.25	8.58	1.09	MSC	1839–1118; 759–399
Fatty acids										
Stearic acid	42	10	97.73	0.23	6.63	61.77	0.79	1.62	1st derivate + SNV	3638–3277; 2559–2198; 1839–1118
Oleic acid	43	10	96.70	0.28	5.51	60.07	0.83	1.59	1st derivate + SNV	3638–3277; 2559–2198; 1839–1118
Linoleic acid	38	8	88.13	1.83	2.90	55.49	3.15	1.50	1st derivate + SNV	3638–3277; 1839–1118
Linolenic acid	43	7	98.47	0.32	8.07	38.36	1.82	1.27	2nd derivate	3278–2918; 2559–2198; 1119–759
Arachidonic acid	40	9	97.03	0.12	5.80	77.67	0.29	2.12	MSC	2919–2558; 1119–759
Vaccenic acid	43	5	93.70	0.01	3.98	67.11	0.01	1.74	1st derivate + SNVi	2559–2199
Docosapentaenoic acid (DPA)	43	10	97.03	0.12	5.80	76.39	0.29	2.06	1st derivate + SNV	3638–3277; 2919–2558; 1839–1118
Eicosapentaenoic acid (EPA)	42	10	87.47	0.13	2.83	54.64	0.22	1.50	None	3104–2501; 2130–893
Docosahexaenoic acid (DHA)	43	3	97.03	0.12	5.80	76.39	0.03	2.06	1st derivate + SNV	3638–3277; 2919–2558; 1839–1118
Total n-3 Polyunsaturated fatty acids (PUFAs)	39	4	90.73	0.58	3.29	41.19	1.37	1.30	2nd derivate	3278–2918; 2559–2198
Total n-6 Polyunsaturated fatty acids (PUFAs)	40	8	94.87	1.46	4.42	73.89	2.90	1.96	MSC	2919–2558; 1119–759
Quality parameters										
Red	42	7	98.06	2.53	7.18	58.47	10.5	1.55	1st derivate + MSC	3998–3637; 1119–759
Green	43	7	91.65	3.24	3.46	70.32	5.55	1.84	1st derivate + MSC	3998–3637; 2198–1118
Blue	39	8	96.03	2.77	5.02	73.63	6.26	1.95	1st derivate + MSC	3998–3637; 2198–1118
L* (Lightness)	46	1	6.80	2.59	1.04	5.28	2.69	1.00	Max. and min. normalisation	3090–2497; 2300–980
a* (Redness)	46	1	25.70	1.76	1.16	15.85	1.83	1.09	Max. and min. normalisation	3200–2500; 2300–980
b* (Blue)	46	1	31.01	2.16	1.20	21.55	2.25	1.13	Max. and min. normalisation	3200–2500; 2300–980
C (Chroma)	46	1	36.74	1.78	1.26	22.60	1.93	1.14	Max. and min. normalisation	3200–2500; 2300–980
h (Hue angle)	46	1	11.71	4.06	1.06	4.49	4.13	1.02	Max. and min. normalisation	3200–2500; 2300–980
Warner–Bratzler shear force (WBSF)	40	3	44.62	10.3	1.34	25.7	11.6	1.60	1st derivate + SNV	1119–759
Tenderness	46	1	36.17	0.49	1.24	27.41	0.50	1.17	Max. and min. normalisation	3200–2500
Juiciness	46	1	18.91	0.56	1.12	8.04	0.58	1.04	Max. and min. normalisation	3200–2500
Overall appraisal	46	1	29.49	0.39	1.19	23.89	0.39	1.05	1st derivate	3200–2500

MSC: Multiplicative Scatter Correction; SNV: Vectorial Normalization; h multiplicative scattering correction; i standard normal variate. a: number of samples used in calibration, b: number of terms included in the equation, c: coefficient of determination for calibration, d: root mean square error of calibration, e: coefficient of determination for cross-validation; f: root mean square error of cross-validation; g: ratio of prediction to deviation;

**Table 3:** Classification matrix from MIR spectrum of 13- and 26-month-old Galician Mountain x Burguete crossbred foals (13M, 26M) and supplemented with standard and linseed concentrate (SC, LC). Discriminant analyses results after selecting the most suitable wavelength range from the prediction models.

**Spectral Wavelength**		**Classify Into**	
Range 3278–2918 cm^−1^			
		13M	26M
13-month-old (%)		63.6	36.4
26-month-old (%)		29.2	70.8
		SC	LC
Standard Concentrate (%)		56.5	43.5
Linseed Concentrate (%)		34.8	65.2
Range 2919–2558 cm^−1^			
		13M	26M
13-month-old (%)		86.4	13.6
26-month-old (%)		37.5	62.5
		SC	LC
Standard Concentrate (%)		--	--
Linseed Concentrate (%)		--	--
Range 2198–1118 cm^−1^			
		13M	26M
13-month-old (%)		77.3	22.7
26-month-old (%)		20.8	79.2
		SC	LC
Standard Concentrate (%)		78.3	21.7
Linseed Concentrate (%)		34.8	65.2

--: Any possible samples’ classification according to finishing diet with that selected range.

## References

[1] Karoui R., Downey G., Blecker C. (2010). Mid-Infrared Spectroscopy Coupled with Chemometrics: A Tool for the Analysis of Intact Food Systems and the Exploration of Their Molecular Structure-Quality Relationships-A Review. Chem. Rev..

[2] de Oliveira G.A., de Castilhos F., Renard C.M.G.C., Bureau S. (2014). Comparison of NIR and MIR spectroscopic methods for determination of individual sugars, organic acids and carotenoids in passion fruit. Food Res. Int..

[3] Lozano M., Rodríguez-Ulibarri P., Echeverría J.C., Beruete M., Sorolla M., Beriain M.J. (2017). Mid-Infrared Spectroscopy (MIR) for Simultaneous Determination of Fat and Protein Content in Meat of Several Animal Species. Food Anal. Method..

[4] Yancey J.W.S., Applea J.K., Meullenetb F.F., Sawyer J.T. (2010). Consumer responses for tenderness and overall impression can be predicted by visible and near-infrared spectroscopy, Meullenet–Owens razor shear, and Warner–Bratzler shear force. Meat Sci..

[5] Ripoll G., Albertí P., Panea B., Olleta J.L., Sañudo C. (2008). Near-infrared reflectance spectroscopy for predicting chemical, instrumental and sensory quality of beef. Meat Sci..

[6] Belaunzaran X., Bessa R.J., Lavín P., Mantecón A.R., Kramer J.K., Aldai N. (2015). Horse-meat for human consumption—Current research and future opportunities. Meat Sci..

[7] Lorenzo J.M., Crecente S., Franco D., Sarriés M.V., Gómez M. (2014). The effect of livestock production system and concentrate level on carcass traits and meat quality of foals slaughtered at 18 months of age. Animal.

[8] Franco D., Lorenzo J.M. (2014). Effect of muscle and intensity of finishing diet on meat quality of foals slaughtered at 15 months. Meat Sci..

[9] Sarriés M.V., Beriain M.J. (2006). Colour and texture characteristics in meat of male and female foals. Meat Sci..

[10] Ruiz M., Sarriés M.V., Beriain M.J., Crecente S., Domínguez R., Lorenzo J.M. (2017). Relationship between carcass traits, prime cuts and carcass grading from foals slaughtered at the age of 13 and 26 months and supplemented with standard and linseed-rich feed. Animal.

[11] Domínguez R., Pateiro M., Crecente S., Ruiz M., Sarriés M.V., Lorenzo J.M. (2018). Effect of linseed supplementation and slaughter age on meat quality of grazing cross-bred Galician x Burguete foals. J. Sci. Food Agr..

[12] Bonnet M., Kopp J. (1986). Dosage du collagène dans les tissus conjonctifs, la viande et les produits carnés. Viandes Produits Carnés.

[13] The American Meat Science Association (AMSA) (2012). Meat color measurement guidelines. American Meat Science Association.

[14] Karamucki T., Jakubowska M., Rybarczyk A., Gardzielewska J. (2013). The influence of myoglobin on the colour of minced pork loin. Meat Sci..

[15] Commission Internationale de l’Eclairage (2008). Colorimetry—Part 4: CIE 1976 L*a*b* Colour Spaces.

[16] Mendizabal J., Purroy A., Indurain G., Insausti K. (2005). Medida del grado de veteado de la carne mediante análisis de imagen. Estandarización de las Metodologías para Evaluar la Calidad del Producto (Animal Vivo, Canal, Carne y Grasa) en los Ruminates.

[17] Beriain M.J., Sánchez M., Carr T.R.A. (2014). Comparison of consumer sensory acceptance, purchase intention, and willingness to pay for high quality United States and Spanish beef under different information scenarios. J. Anim. Sci..

[18] The American Meat Science Association (AMSA) (2015). Research Guidelines for Cookery, Sensory Evaluation, and Instrumental Tenderness Measurements of Meat. Research Guidelines for Cookery, Sensory Evaluation, and Instrumental Tenderness Measurements of Meat. American Meat Science Association.

[19] Macfie H.J., Bratchell N., Greenhoff K., Vallis L.V. (1989). Designs to Balance the Effect of Order of Presentation and First-Order Carry-Over Effects in Hall Tests. J. Sens. Stud..

[20] Martens H., Naes T. (1989). Multivariate Calibration.

[21] Jović O., Smolić T., Jurišić Z., Meić Z., Hrenara T. (2013). Chemometric Analysis of Croatian Extra Virgin Olive Oils from Central Dalmatia Region. Croat. Chem. Acta.

[22] Lucarini M., Durazzo A., Sánchez del Pulgar J., Gabrielli P., Lombardi-Boccia G. (2018). Determination of Fatty Acid Content in Meat and Meat products: The FTIR-ATR approach. Food Chem..

[23] Vlachos N., Skopelitis Y., Psaroudaki M., Konstantinidou K., Chatzilazarou A., Tegou E. (2006). Applications of Fourier transform-infrared spectroscopy to edible oils. Anal. Chim. Acta.

[24] Rohman A., Sismindari E.Y., Che Man Y.B. (2011). Analysis of pork adulteration in beef meatball using Fourier transform infrared (FTIR) spectroscopy. Meat Sci..

[25] Oliván M., de La Roza B., Mocha M., Martínez M.J. Prediction of physico-chemical and texture characteristics of beef by near infrared transmittance spectroscopy. Proceedings of the 10th International Conference on Near Infrared Spectroscopy.

[26] Lanza E. (1983). Determination of moisture, protein, fat and calories in raw pork and beef by near infrared spectroscopy. J. Food Sci..

[27] Shi H.T., Lei Y.G., Prates L.L., Yu P.Q. (2019). Evaluation of near-infrared (NIR) and Fourier transform mid-infrared (ATRFT/MIR) spectroscopy techniques combined with chemometrics for the determination of crude protein and intestinal protein digestibility of wheat. Food Chem..

[28] Cozzolino D., Murray I., Scaife J.R., Paterson R. (2000). Study of dissected lamb muscles by visible and near infrared reflectance spectroscopy for composition assessment. Anim. Sci..

[29] Weeranantanaphan J., Downey G., Allen P., Sun D.-W. (2011). A review of near infrared spectroscopy in muscle food analysis: 2005–2010. J. Near Infrared Spectrosc..

[30] Dilzer A., Park Y. (2012). Implication of Conjugated Linoleic Acid (CLA) in Human Health. Crit. Rev. Food Sci. Nutr..

[31] Geesink G.H., Schreutelkamp F.H., Frankhuizen R., Vedder H.W., Faber N.M., Kranen R.W., Gerritzen M.A. (2003). Prediction of pork quality attributes from near infrared reflectance spectra. Meat Sci..

[32] Andrés S., Murray I., Navajas E.A., Fisher A.V., Lambe N.R., Bünger L. (2007). Prediction of sensory characteristics of lamb meat samples by near infrared reflectance spectroscopy. Meat Sci..

[33] Hell J., Prückler M., Danner L., Henniges U., Apprich S., Rosenau T., Kneifel W., Bohmdorfer S. (2016). A comparison between near-infrared (NIR) and mid-infrared (ATR-FTIR) spectroscopy for the multivariate determination of compositional properties in wheat bran samples. Food Control.

[34] Xing J., Ngadi M., Gunenc A., Prasher S., Gariepy C. (2007). Use of visible spectroscopy for quality classification of intact pork meat. J. Food Eng..

[35] Juárez M., Alcalde M.J., Horcada A., Molina A. (2008). Southern Spain lamb types discrimination by using visible spectroscopy and basic physicochemical traits. Meat Sci..

